# Improving cancer prevention and control through implementing academic-local public health department partnerships – protocol for a cluster-randomized implementation trial using a positive deviance approach

**DOI:** 10.1186/s43058-025-00706-z

**Published:** 2025-02-24

**Authors:** Stephanie Mazzucca-Ragan, Peg Allen, Kathleen Amos, Abigail R. Barker, Madisen Brewer, Paul C. Erwin, Jessica Gannon, Feng Gao, Rebekah R. Jacob, Rebecca Lengnick-Hall, Ross C. Brownson

**Affiliations:** 1 Brown School at Washington University in St. Louis, St. Louis, MO USA; 2 Public Health Foundation, Washington, DC USA; 3 School of Public Health, The University of Alabama at Birmingham, Birmingham, AL USA; 4 Division of Public Health Sciences, Department of Surgery and Alvin J. Siteman Cancer Center, Washington University School of Medicine, Washington University in St. Louis, St. Louis, MO USA

**Keywords:** Cancer prevention and control, EPIS, Public health, Bridging factors, Positive deviance

## Abstract

**Background:**

Local public health departments in the United States are responsible for implementing cancer-related programs and policies in their communities; however, many staff have not been trained to use evidence-based processes, and the organizational climate may be unsupportive of evidence-based processes. A promising approach to address these gaps is through academic-public health department (AHD) partnerships, in which practitioners and academics collaborate to improve public health practice and education through joint research projects and educational opportunities. Prior research has demonstrated the benefits of AHD partnerships to public health practice and education. However, knowledge about how AHD partnerships should be structured to support implementation of programs and policies is sparse.

**Methods:**

This is a mixed methods, two-phase study, guided by the Exploration, Preparation, Implementation, and Sustainment (EPIS) Framework, in which AHD partnerships are a relational type of bridging factor. A positive deviance approach will be used to understand how AHD partnerships are best structured and supported. In the formative phase, we will survey academics and local health department staff (*n = *500) to characterize AHD partnerships and understand contextual influences. We will conduct in-depth interviews with eight AHD partnerships (four high and four low engagement), to identify differences between high and low engagement partnerships. The second, experimental phase will be a paired group randomized trial with 28 AHD partnerships (*n = *14 randomized to implementation arm and *n = *14 to the control arm). A menu of strategies will be refined through survey and interview findings, literature, and our team’s previous work. The trial will assess whether these strategies can be used to strengthen partnerships and improve adoption of cancer prevention and control programs and policies. We will evaluate changes in AHD partnership engagement and implementation of evidence-based programs and policies.

**Discussion:**

This first-of-its-kind study will focus on collaborations that leverage complementary expertise of health department staff and academics to improve public health practice. Our results can impact the field by identifying new, sustainable models for how public health practitioners and academics can work together to meet common goals, increase the use of evidence-based programs and policies, and expand our understanding of bridging factors within the EPIS framework.

**Trial registration:**

Prospective registered on 9/17/2024 at clinicaltrials.gov no. NCT06605196 (https://clinicaltrials.gov/study/NCT06605196).

Contributions to the literature

- This study examines academic-health department (AHD) partnerships, which are an underutilized model for how public practice and academic public health can work together to meet common goals, importantly the implementation of evidence-based programs and policies. This will be the first study to experimentally test implementation strategies to support AHD partnerships.
- AHD partnerships are a relational type of bridging factor, an emerging component of the EPIS framework. This study will expand our understanding of bridging factors and methodologies for how to study them.
- The use of a positive deviance approach as part of the process of selecting implementation strategies is a useful methodology to capture local knowledge and solutions among successful partnerships and ensure that implementation strategies are appropriately chosen based on the contextual factors within a partnership.

## Background

Cancer is the second leading cause of death in the United States (US) [1]; however, between one-third and one-half of deaths due to cancer are preventable [2–80]. By applying evidence-based programs and policies (EBPPs) that focus on improving modifiable risk factors, such as diet, physical activity, tobacco use, and screening and early detection, the incidence of cancer and impacts on healthcare systems can be reduced [2, 5–8]. National-level efforts (*e.g.,* the *Community Guide*) highlight a variety of EBPPs that cancer control practitioners can implement in their communities [9–11]. Also, approaches such as evidence-based public health, *i.e.,* the process of integrating science-based interventions with community preferences to improve the health of populations [12], are available to and recommended for public health professionals to successfully adopt and implement EBPPs into real-world public health practice [12–16]. However, EBPPs are used inconsistently among practitioners [12, 17]. One study found that less than half of cancer control planners had ever used evidence-based resources [18], which indicates that additional, active strategies are needed to ensure that public health professionals effectively use the resources available to them and implement EBPPs.

An important group of public health professionals are the practitioners in the 2,800 US local health departments (LHDs), who are on the “front lines” of public health.

LHDs work at the city, county, or regional level and provide two-thirds of all public health activities [19–22]. LHD activities and expenditures are associated with reduced deaths from diabetes, heart disease, and cancer [23]. LHDs are ideally positioned to address chronic diseases because of their particular strengths to assess a public health problem within the communities they serve and cultivate partnerships needed to implement an EBPP [24–27]. Activities such as reviewing the best available peer-reviewed evidence, using data, applying program planning frameworks, and conducting sound evaluation are among those most important for evidence-based public health to occur [12]. For many LHD practitioners, skills to conduct these activities are lacking [28, 29]. Organization-level structures and processes (*e.g.*, leadership, organizational climate and culture, access to research information) need to be present [12, 15, 30, 31, 81, 82]. Yet, barriers to utilizing evidence-based practices exist, such as lack of incentives, inadequate connections between research and practice, and absence of cultural and leadership support [14, 32–34, 82].

In the last two decades, there has been a growing recognition of academic-health department (AHD) partnerships as a strategy to improve public health practice and education [35–38]. These partnerships offer an existing structure to deliver the training, technical assistance, and other supports to LHDs. AHD partnerships are a type of community-academic partnership, defined as “an arrangement between an academic institution and a governmental public health agency that provides mutual benefits in teaching, research, and service” [39]. Academicians can improve the real-world relevance of their research and teaching, and public health practitioners and agencies can receive support to implement EBPPs. Erwin and colleagues found that LHDs engaged in an AHD partnership were 2.3 times more likely to implement EBPPs compared to LHDs with no AHD partnership [40]. The arrangements can be formal (*e.g.,* through a memorandum of understanding) or informal and provide collaborative opportunities across academia and practice [41, 42]. These partnerships are common; in the 2022 Profile of Local Health Departments, 89% of LHDs reported engagement with schools or programs of public health and 64% reported formal written agreements [43].

To our knowledge, no studies have systematically examined the structures and processes within AHD partnerships that support EBPP implementation or used experimental designs to understand what is needed for AHD partnerships to support EBPP implementation. This is a critical gap as AHD partnerships represent a “relational tie” type of bridging factor [44]. Bridging factors are an emerging construct in the Exploration, Preparation, Implementation and Sustainment (EPIS) framework [45]. This study advances bridging factors research in two critical ways. First, it advances the methodology of studying bridging factors because it will utilize a suite of methods to describe AHD partnership characteristics and isolate what features of these partnerships can be intervened upon through the use of implementation strategies. Second, the implementation strategies that arise and will be tested in this study will directly support the way that these AHD partnerships function to support EBPP implementation and sustainment. Findings can build implementation strategy knowledge around this commonly used relational tie type of bridging factor.

Our specific aims are as follows:

- Aim 1: Understand the structures of and processes within AHD partnerships, and the contextual factors that influence the ability of AHD partnerships to implement EBPPs.
- Research Questions: How do successful AHD partnerships build and maintain individual skills and organizational capacity required to implement and evaluate implementation of EBPPs? What contextual factors promote or hinder a successful AHD partnership?
- Aim 2: Test the effectiveness of strategies designed to increase the implementation of EBPPs for cancer prevention and control by strengthening AHD partnerships.
- Hypothesis: Two years post randomization, LHDs within partnerships randomized to receive supports to improve AHD partnerships will have implemented significantly more cancer-related EBPPs compared to LHDs within partnerships randomized to the control arm.

## Methods/design

### Overview of study design

This is a mixed methods implementation science study that consists of two complementary phases. The formative phase focuses on refining our understanding of AHD partnerships and the structures and processes that may contribute to the ability of a partnership to facilitate EBPP implementation. The experimental phase uses a paired group-randomized trial to test the effectiveness of strategies to increase EBPP adoption by improving AHD partnerships. The study uses a positive deviance approach and is guided by the Exploration, Preparation, Implementation, and Sustainment (EPIS) framework [45, 46].

### Exploration, Preparation, Implementation, and Sustainment (EPIS) framework

The work is guided by the EPIS framework, which is a process and determinant framework [47] that identifies four phases of the implementation process: *exploration* of the health needs and the best available EBPP to address them; *preparation* for potential barriers and facilitators during implementation and necessary adaptations to the EBPP; *implementation* and evaluation of the EBPP; and *sustainment* of the EBPP [48]. The framework highlights determinants in the outer context, *i.e.,* the public health and policy environment and characteristics of EBPP recipients, and inner organizational context of LHDs, such as leadership, organizational structures, and procedures, on the implementation process [45, 48]. Features of an EBPP itself, including the fit of the EBPP with its recipients and the implementing organizations, may also influence implementation. This study offers an opportunity to deepen our knowledge of the processes and determinants outlined in the framework and understand how they operate in different systems. A recent addition to EPIS is the concept of bridging factors, which are structures and processes that cross and connect the outer system and inner organizational context and influence the implementation process [45]. Bridging factors include community-academic partnerships, such as AHD partnerships of inquiry in this study, intermediary organizations that provide support for EBPP implementation, and formal arrangements and processes (*e.g.,* contracts) [45, 49]. Using EPIS and the concept of bridging factors in this study will allow us to understand the activities within partnerships that could be modified to support EBPP implementation [44, 45, 49], i.e., how AHD partnerships should be structured and what processes and resources should be used within an AHD partnership to enhance the adoption of EBPPs [49]. This study provides a rich context for identifying specific ways that LHDs and academic partners influence each other and exchange key resources between the inner context of an LHD and the outer setting to influence EBPP adoption, implementation, and sustainment.

### Positive deviance methodology

The overall methodological approach for the project is the positive deviance methodology, which acknowledges that solutions to problems within a community (here, public health practitioners and researchers) often exist within that community, and the knowledge and experiences of certain members can be generalized to improve the engagement of other members [46]. The positive deviance approach accomplishes two goals – identifying practices associated with top engagement and promoting the use of these practices within a community, using a mixed methods approach [50]. Four steps are used within a positive deviance approach [46]. First is to identify positive and negative deviants, *i.e.,* AHD partnerships that demonstrate high or low implementation of cancer-related EBPPs. Next, organizations identified as positive and negative deviants are studied in-depth using qualitative methods to understand which structures and processes enable AHD partnerships to support implementation of EBPPs and how our existing strategies need to be refined to fit within AHD partnerships. Third, hypotheses generated from the qualitative work are quantitatively tested in a broader sample of partnerships. Last, results are disseminated with input from our practice partners.

### Research setting and partners

This study will focus on AHD partnerships identified through the Council on Linkages Between Academia and Public Health Practice’s Academic Health Department Learning Community, hereafter the Learning Community. The Learning Community is a national network designed to support the development, maintenance, and growth of AHD partnerships [51]. The Learning Community is comprised of members who are interested in sharing knowledge and experiences related to AHD partnerships and creating resources and tools. Members include LHD directors and staff and academicians (in public health or related fields). The Learning Community hosts resources to support AHD partnerships and opportunities for interaction, *e.g.,* meetings, webinars, and online discussions. To date, these resources have focused on partnership building and public health education, but not specifically on how AHD partnerships can support EBPP implementation.

We will engage additional research partners through a Practice Advisory Group that will 1) provide overall project guidance; 2) review data collection methods and instruments; 3) give input on refining the strategies used in the experimental phase; and 4) assist in disseminating findings. The group includes members of national public health practice organizations, public health practitioners, and practice-focused academics, all with expertise in AHD partnerships.

### Formative phase: learn from existing AHD partnerships

This phase focuses on the first two steps of the positive deviance approach: 1) survey AHD partnerships to identify high and low engagement AHD partnerships based on their adoption of cancer-related EBPPs and 2) study these partnerships in depth using qualitative methods (interviews and document reviews).

#### Step 1. Identify positive and negative deviants

We will survey AHD partnerships to identify positive and negative deviants, *i.e.,* AHD partnerships that have high or low adoption of cancer-related EBPPs, and collect data on other AHD outcomes for public health research and practice according to the logic model by Erwin et al. [41].

#### Sampling and recruitment

We will survey LHD practitioners and their academic partners to assess the adoption of cancer-related EBPPs and quantify other measures of partnership success according to a composite measure described below, theoretically guided by the AHD partnership logic model [41]. Using a modified snowball sampling approach, we will invite members of the Learning Community to participate in the survey and will ask those participating to identify other individuals in their partnership. These additional individuals will be invited to participate in the survey. LHD practitioners and their academic partners will be eligible to participate in the survey. We anticipate 500 responses from 100 AHD partnerships (average of 5 respondents per partnership).

#### Measures

The survey is designed to assess engagement related to implementation of cancer prevention and control EBPPs (Table 1) [11, 52]. Adoption of EBPPs will be the variable used to characterize positive and negative deviants and is the primary outcome for the trial. Respondents are presented with a list of evidence-based programs and policies taken from the *Community Guide*, reflecting the program areas in which respondents work. EBPPs are focused on primary or secondary prevention of cancer, grouped into categories used in the *Community Guide*: healthy weight management, physical activity promotion, healthy eating promotion, tobacco use prevention and/or cessation, HPV vaccination, cancer screening, health equity/social determinants of health, maternal and child health, and environmental health [11]. EBPPs that are recommended with sufficient or strong evidence from the Preventive Services Task Force Review are included in the survey. Respondents indicate whether they currently implement (i.e., have adopted) a given EBPP, and a summary variable will be created to reflect adopted EBPPs. This item has been used in our previous work and has demonstrated sufficient test–retest reliability [63]. We will also assess well-recognized outputs and outcomes of successful, productive AHD partnerships [41]. Quantitative indicators include: AHD partnership structures and activities; AHD partnership strengths; organizational supports for evidence-based practice; delivery of evidence-based interventions to address chronic diseases; individual public health skills regarding evidence-based public health; leadership and organizational characteristics; and sustainability of public health programs.

**Table 1:** Survey measures

Survey section	Number of items	Type of variables	Sample items	Item sources
Screening questions	2	Check one, yes/no/unsure, check all	Primary organization, participation in an AHD partnership	New
Academic-Health Department Partnerships	16	Check all, Likert 5-point scale, yes/no,	Characteristics of AHD partnership, training topics addressed by partnership, practice-based research	New, Public Health Workforce Interests and Needs Survey, Brewster 2019, Chen 2008
Interventions to address chronic diseases	23	Check all, yes/no/unsure, open response	Delivery of evidence-based interventions by organization and by AHD partnership	New, Erwin 2019, the Community Guide
Individual skills	14	Likert 5-point scale	Individual public health skills, AHD Partnerships Logic Model assumptions	NACDD survey, Erwin 2019, Padek 2021
Leadership and organizational characteristics	28	Likert 5-point scale, check all, yes/no/unsure	Leadership within agency, characteristics of organization, supervision-related training for new supervisors	Directors Assessment of Workforce Needs Survey, NACDD survey
Sustainability	11	Likert 5-point scale	External support (3), funding stability (5), Strategic planning (3)	Schell 2013
Background	12	Check one, check all, yes/no, open response	Position, participation in academic health professions institution, primary academic appointment, program area, years in position, years in public health, degree/credentials, gender, race, ethnicity, age, accreditation status, names of partner organizations	NACDD survey, New, Public Health Workforce Interests and Needs Survey, Pew Research Center

*AHD* academic health department partnership

#### Survey refinement

The study team and our research and practitioner colleagues have reviewed survey drafts to make sure it can be easily understood by respondents and accurately captures relevant experiences or opportunities. We conducted cognitive response testing with approximately 20 AHD partnership members (practitioners and academics), to improve the quality of data collection [53–56]. Testing was complete when survey items were clear and captured relevant information according to interviewees, and survey items were revised iteratively based on results of the cognitive response testing.

#### Quantitative data collection

Pre-invitation emails will be sent to participants to inform them of the survey purpose, and invitation emails with the survey link will be sent one week later. The survey will take no more than 20 min to complete and will be programmed for data collection in Qualtrics, an online survey software. Those who have not completed the survey will receive up to three reminder emails and two phone calls over a six-week period to address questions about the study and encourage participation. The study timeline is shown in Table 2.

**Table 2:** Study timeline

	**2023**				**2024**				**2025**				**2026**			
	**Q1**	**Q2**	**Q3**	**Q4**	**Q1**	**Q2**	**Q3**	**Q4**	**Q1**	**Q2**	**Q3**	**Q4**	**Q1**	**Q2**	**Q3**	**Q4**
**Aim 1:**																
Quantitative data collection and analysis				X	X											
Qualitative data collection and analysis					X	X	X									
Refine strategies for implementation trial				X	X	X	X									
**Aim 2:**																
Baseline data collection							X	X								
Implement strategies to improve EBPP adoption								X	X	X	X	X	X	X	X	
Process evaluation								X	X	X	X	X	X	X	X	
Follow-up data collection (2 years post- randomization)															X	X

#### Data analysis

Data analysis will be conducted in SAS (version 9.4, Cary, NC). Summary measures will be created separately for all domains by adding items within a domain. For use within the qualitative interviews, an average partnership-level score will be created for each summary measure. Partnerships will be ranked according to the average number of EBPPs reported by partnership members. The four highest- and four lowest-ranked AHD partnerships, *i.e.,* partnerships implementing the most and fewest cancer-related EBPPs, will be considered to have the highest and lowest engagement and will be invited to participate in the subsequent step 2. In the event of a tie, we will randomly choose one partnership to invite.

### Step 2. Study positive and negative deviants

Individual interviews will be used to study positive and negative deviants in depth. Once a group of positive and negative deviants is identified in step 1, LHD practitioners and their academic partners in positive and negative deviant AHD partnerships will be invited to participate in qualitative, key informant interviews and provide information for document reviews. Interviews and document reviews will yield data to understand the extent to which AHD partnerships operate, as a bridging factor, to contribute to EBPP implementation, *i.e.,* the structures, processes, and resources used within a partnership to connect the inner and external contexts. Data from this step will prioritize what strategies are used in the experimental phase to enhance AHD partnerships to improve EBPP implementation.

#### Sampling, recruitment, and interview domains

We will use a purposive, snowball sampling approach to identify key informants for interviews [57]. This approach allows us to identify those who have first-hand knowledge about the AHD partnerships from the perspective of LHD staff and academics. For partnerships identified as a positive or negative deviant, we will invite the survey respondents from step 1 to participate in an interview. Similar to the approach to identify other respondents for the quantitative survey, at the end of the interview we will ask the respondent to name individuals within the partnership (*i.e.,* practitioners and academics). An average of five participants per AHD partnership will be recruited, including three participants representing the LHD (total recruitment: 8 AHD partnerships × 5 participants/partnership = 40 participants).

The interviews will take an ontological approach and a pragmatic interpretive framework, as we will seek to understand what is useful, practical, and works from the perspectives of more and less successful AHD partnership members [58]. The interviews will focus on several domains: 1) how the partnership formed; 2) how it has been maintained; 3) key characteristics of the partnership; 4) relevant details about the outer context, especially those related to funding and policy; and 5) how well the structures, processes, and resources within the partnership bridge the inner and outer contexts to support EBPP implementation.

#### Document review

In addition to qualitative interviews of those in successful AHD partnerships, we will collect formal documents that provide guidance about how successful AHD partnerships are structured. Documents will be requested at the conclusion of the interview and will be shared digitally with the study team. Example documents are Memoranda of Understanding (MOU), which outline the terms of a partnership, project reports from joint research projects, and accreditation documents, which often are required to have information about EBPP implementation and practice-based education (student practica).

#### Qualitative data and document review coding and analysis

Digital recordings will be transcribed verbatim by the Internet-based service Rev (https://www.rev.com/). All transcripts and documents will be analyzed using NVIVO.12 software [59]. A codebook will be developed based on the interview guide, which will be based on EPIS. Each transcript will be coded independently by two team members [60]. The two team members will then review non-overlapping coding in the text blocks and reach agreement on text blocking and coding. Themes from the coded transcripts and documents will be summarized and highlighted with exemplary quotes or in data matrices.

#### Refining strategies to enhance AHD partnership engagement and increase use of EBPPs

A list of AHD partnership features (*e.g.,* characteristics, structures) and processes used to form or maintain AHD partnerships that may contribute to EBPP implementation will be compiled based on emerging themes from the qualitative interviews and document reviews. To create an initial menu of strategies, we will match these features to strategies used in prior work [61–65] or in the Learning Community using a nominal group technique, a structured variation of a small-group discussion, to reach consensus [66]. To refine this list, the investigative team and Practice Advisory Group will review themes to prioritize those that are most likely to be influential for EBPP implementation, as some features may be influential for other outcomes of AHD partnerships such as student practica. We will build consensus as a group using prioritization matrices commonly employed in small group decision making and prioritization activities [67–69]. We will rate partnership features according to characteristics such as appeal, perceived relevance to EBPP implementation, feasibility, cost, and sustainability potential and prioritize based on the combinations of these ratings.

### Experimental Phase: test strategies to improve EBPP implementation through AHD partnerships (positive deviance step 3)

In the experimental phase, we will test strategies to support AHD partnerships and increased use of EBPPs. To test the hypotheses generated in the formative phase, we will conduct a paired, group-randomized study to understand if the strategies used within top engagement AHD partnerships can improve adoption of cancer prevention and control EBPPs among lower engagement AHD partnerships. Fourteen partnerships will be randomized to the implementation arm, which will receive resources and guided facilitation, and 14 will be randomized to a control arm, which will be referred to existing resources in the Learning Community but will not receive new resources or the guided facilitation.

#### Study population and selection of AHD partnerships

Our target audiences for this study are LHD practitioners and academics in lower engagement AHD partnerships as defined by extent of LHD EBPP implementation. We will recruit participants for this study from those identified as the bottom half of the EBPP implementation score created in the formative phase. Randomization will occur after pair matching as in previous community trials, to improve power [70–72]. Matching of partnerships will be based on size of the LHD and how long the AHD partnership has been in existence. Individuals from the LHD and academic institution will be invited to participate in the study. We will recruit 13 LHD staff and academic partners per partnership. LHD staff will include the division or program director for community health promotion or chronic disease prevention (primary contact) along with staff identified by the program director key to supporting cancer prevention and control, *e.g.,* health educators. Academic partners will include professors, research staff, and graduate students from schools or programs of public health, nursing, or other related health disciplines. To increase achievability of the primary outcome, we will limit our sample to LHDs with at least 2 FTEs focused on chronic disease control per our prior research [63]. This provides a total of 364 (28 partnerships X 13 partnership members/partnership) individuals for recruitment into the trial.

#### AHD partnership strengthening strategies

AHD partnerships randomized to receive remote tailored, guided support to improve their partnerships will first participate in a planning period at the beginning of the intervention period to establish the goals for the partnership (Table 3). The study team will facilitate discussions to determine how AHD partnerships want to modify their partnership. We will use the prioritized list of AHD partnership features developed in the formative phase to guide the initial planning period and then assist the partnership members to plan to implement the corresponding strategies focused on their areas of interest. This approach acknowledges that uniformity is unlikely to be effective across partnerships with different needs, capacity, and context [73–76].

**Table 3:** Implementation strategy description

	**Strategy: Assess for readiness and identify barriers and facilitators**	**Strategy: Tailored facilitation**
**Actor(s)**	Study staff trained in public health and who have experience working with governmental public health staff	Study staff trained in public health and who have experience working with governmental public health staff
**Action(s)**	Initial videoconference meeting to understand the goals for the partnership, discuss barriers and facilitators, make suggestions, and provide encouragement	Ongoing videoconference calls to reflect on the progress towards goals and the implementation effort, share lessons learned, work through challenges, and discuss any changes that need to be made to goals. Additional implementation strategies (e.g., access new funding, conduct ongoing training) may be recommended for use to match goals
**Target(s) of the action**	Local health department staff and their academic partners	Local health department staff and their academic partners
	Knowledge about and skills to use evidence-based programs and policies, understanding of each partner’s strengths to support implementation	Knowledge about and skills to use evidence-based programs and policies, understanding of each partner’s strengths to support implementation
**Temporality**	The initial meeting will be within the first month of the implementation period	First facilitation meeting should be within one month of initial goal setting meeting
**Dose**	Once for one hour	At least once monthly for up to one hour for the study period (2 years)
**Implementation outcome(s) affected**	Adoption of cancer-related, evidence-based programs and policies	Adoption of cancer-related, evidence-based programs and policies
**Justification**	Local health department practitioners often lack skills to implement evidence-based programs [28, 29]. Understanding context-specific barriers and facilitators to utilizing evidence-based practices is key to supporting change within local health departments [14, 32–34, 82].	Tailoring is essential because uniformity is unlikely to be effective across partnerships with different needs, capacity, and context [73–76].

Description categories taken from Proctor et al. 2013 [83]

Once the initial planning period is complete, the research team will be knowledge brokers to provide facilitation and improve AHD partnership engagement in ways that match the goals set by the partnership. Based on our prior work, we anticipate LHD staff will need guided facilitation and training focused on: 1) assistance with strategic planning processes for implementing a strategy of interest; 2) consultation on overcoming barriers to implementing EBPPs; 3) help identifying funding sources and grant writing to fund joint research projects; and 4) identification of online training sources to support evidence-based decision making and strengthen partnerships. Instead of providing these supports directly to LHD staff as in previous work, we will work with the AHD partnership to meet these needs. We will regularly assess AHD partnerships’ progress towards goals, and adjust goals and strategies based on the progress.

#### Quantitative measures

To understand the effects of AHD partnership strengthening activities, we will measure the adoption of cancer-related EBPPs (primary outcome) and other partnering, individual- and organizational-level covariates. Data will be collected at baseline (prior to randomization) and at the end of the study.

#### AHD partnership engagement

Measures will include the same collected in the formative phase, including the adoption (use) of cancer prevention and control EBPPs (primary outcome); AHD partnering activities; organizational supports for evidence-based decision making [77]; joint research projects; publishable practice-based research; shared staff; and shared financial resources.

#### Implementation costs

Given the importance of cost in public health decision making and for future scale up and replication studies [78–80, 84, 85], costs associated with various strategies will be collected using a pragmatic approach proposed by Cidav and colleagues [86]. The approach blends time-driven activity-based costing, a process-based micro-costing method used in business accounting, with Proctor’s framework for reporting implementation strategies [86]. Costs are tracked in a step-wise manner: 1) name each implementation strategy and list the associated actions, actors, and temporality; 2) determine the frequency and average duration of each implementation action by actors and actors’ total time spent on each action; 3) determine the price per hour of each actor; 4) determine non-personnel, fixed resources and their expenses; and 5) calculate total costs. This approach will allow us to describe the relationship between costs and outcomes, both cumulatively and by implementation action. Costs associated with research activities will be separated from other implementation costs.

#### Organizational covariates

Information about the AHD partnership will be collected, including the year the partnership was established, the number of individuals involved in the partnership, and bridging factor dimensions of the partnership (*e.g.,* partnership resources and structures to support EBPP implementation). Characteristics of the LHD (FTEs, annual expenditures, jurisdiction size, Public Health Accreditation Board accreditation status) and academic institutions (2- or 4-year institutions, Public Health Schools or Programs) will be collected.

#### Individual covariates

LHD staff and academicians’ age group, years in current position, job title, and academic degrees will be collected. Previously validated surveys will assess LHD staff’s evidence-based decision making skills [61, 87, 88].

#### Qualitative measures

Participants in partnerships randomized to the active implementation arm will be invited to participate in a post-study interview to understand how and why changes did or did not occur, according to EPIS framework constructs that may explain the quantitative findings in the study. Similar to the formative phase, interview questions will assess how partnerships were structured and what processes and resources AHD partnership members thought were most and least impactful for enhancing the adoption of EBPPs.

#### Process evaluation

Process evaluation data will assist us in determining the use and reach of our strategies and to assess whether outside events impacted our findings. Examples of process measures include requests for technical assistance, participation and satisfaction with capacity building initiatives, inclusion of a greater focus on EBPH and EBPPs in agency plans, becoming an accredited or reaccredited health department, and external funding granted to an LHD for cancer control-related programming.

#### Power calculation

This study uses a paired, group randomized design. By using tight matching criteria, we will balance potential confounding factors (*e.g.*, FTEs, county size, duration of partnership) that may affect EBPP implementation. As a result, the between-cluster variation will be reduced, and statistical power increases [89, 90]. Based on our preliminary studies and values of intraclass correlation coefficients (ICC) in the literature [30, 62, 91–96], we estimated a range of effect sizes and ICCs. We calculated a median ICC from similar studies and developed a range based on a 50% decrease and increase around the median (range 0.009 to 0.027). We are interested in the changes in AHD partnership characteristics and adoption of EBPPs from baseline between the intervention and control arms. Drawing from previous work [63, 92, 93], we hypothesize that the scores in the intervention arm will be between 10 and 20% higher than the control arm for implementation of EBPPs. Following Donner [97] and Thompson [98], and using the most conservative estimates of effect sizes and ICCs, we estimate the number of clusters needed is 28 AHD partnerships (*n = *14 per arm) and the number of subjects is 13 at baseline (total = 364) to ensure 9 at two years post-randomization (total = 252), given a power of > 90% and an overall Type I error of 5%. This assumes about a 70% retention rate of subjects and allows for attrition of one cluster.

### Quantitative analyses

We will follow Donner [97] for analysis of matched-pair quantitative data. We consider the difference in the mean change between intervention and control EBPP implementation scores in an AHD partnership pair as the unit of analysis and will use the weighted paired t-test (with the cluster size as weights) if the cluster size varies across pairs. We will use the permutation test in which we compare the observed difference with the null distribution derived from the permutation procedure. For cluster-level adjusted analyses, we will follow Thompson’s approach [98] to obtain the adjusted mean first for each individual and for each partnership, then the adjusted difference in mean change in a pair. Modeling details are available upon request.

#### Qualitative analyses

Interview data will be analyzed using the same approach outlined for the interviews conducted in the formative phase.

#### Mixed methods analyses

In the formative phase, the quantitative and qualitative data will be collected and analyzed as a concurrent, embedded design (qual + QUAN), in which the qualitative findings will elaborate upon the quantitative findings to triangulate results, according to accepted best practices [99, 100]. The two types of data will be connected such that the qualitative data builds off of the quantitative data and analyzed with complementarity in mind, which will allow us to elaborate upon quantitative findings [99]. We will use a social constructivist approach and ontological assumptions during data analysis, as our emphasis will be on understanding AHD partnerships from the perspectives of partnership members (*i.e.,* LHD staff and academic partners), acknowledging that there are likely different experiences for each AHD partnership [58]. Data can be integrated in a joint results display to visualize linkages between the quantitative and qualitative data [50].

### Step 4. Disseminate findings

To complete the positive deviance approach, we will use a multi-component, active dissemination strategy to communicate findings [73, 101, 102]. Our systematic approach will be guided by principles of Designing for Dissemination (D4D) to effectively reach all relevant groups [103–106]. D4D applies the concept of audience segmentation, acknowledging that communication with different audiences requires messages and channels specific to their needs and preferences [107–110]. Keeping a focus on dissemination from the beginning of the project ensures that our findings are useful, relevant, and ready for dissemination before funding ends.

Because we will have LHD practitioners and academics as members of our Practice Advisory Group, we will have natural advocates for project dissemination. Our Practice Advisory Group will: 1) review the initial findings from the project phases especially in regard to their relevance for practitioners; 2) identify resources for enhancing the reach of the project; and 3) identify opportunities for disseminating project findings. LHD practitioners and their academic partners will be asked to provide input on formats and channels for dissemination materials (*e.g.,* reports, webinars, toolkits, social media platforms).

Strategies will then be employed according to the target audience. Scientific researchers will be reached via publications in high-impact, practice-oriented peer-reviewed journals and research meetings. Public health practitioners will be reached through a combination of channels, including our partner organizations which regularly communicate with their members through email and webinars. All materials from our project (key findings, survey instruments) will be posted on our center website. A particular emphasis will be placed on social media (*e.g.,* LinkedIn, Facebook), as these platforms are increasingly important in communicating to practitioners and researchers [111, 112].

## Discussion

This study will contribute to the public health-focused implementation science literature in several ways. To our knowledge this is the first experimental, longitudinal study focused on local-level collaborations that leverage the expertise of LHDs and academics to improve public health practice. Also, this study will advance bridging factors research [44, 45, 49], specifically refining methods used to describe bridging factors and how they influence EBPP implementation, identifying modifiable aspects of bridging factors, and testing implementation strategies to strengthen bridging factors. Third, this study examines new models for how public health practice and academic public health can work together to meet common goals. The Institute of Medicine (now the National Academy of Medicine) noted the potential benefits of community-academic partnerships for public health education and practice [26], and this study could make the recommendations in these reports a reality. Lastly, a project on the scale intended here has the potential to begin to shift the paradigm on how research on EBPPs can be more quickly and effectively translated to those in the best position to use the evidence (here, LHD practitioners) and how public health research and practice can be efficiently funded to improve public health outcomes.

There are several potential limitations to this study. First, many AHD partnerships enhance workforce development broadly through activities such as internship programs and training programs across multiple topics and may or may not specifically address cancer prevention and control. Second, while strategies AHD partnerships employ may strengthen the partnership overall, it may take more time than the allotted intervention period to impact cancer control EBPP implementation. Third, the number of cancer control EBPPs implemented by LHDs as the main outcome variable is a narrow parameter, that while measurable, might not demonstrate enhanced implementation quality, sustainability, impact, or strength of the LHD partnerships.

## Conclusion and impact

AHD partnerships play a critical role in advancing the implementation of EBPPs [40]. By learning about the way in which academic-health department partnerships work and the effectiveness of strategies in supporting them, we can identify relevant, effective capacity building and implementation strategies to increase local capacity for cancer prevention and control and other, similar EBPPs. More broadly, knowledge from this study can inform future work involving the assessment of or intervention on bridging factors. The implementation strategy cost information we will collect may be useful not only to LHDs and AHD partnerships seeking to increase EBPP uptake, but also to inform other goals in AHD partnerships and public health practice. Our results have the potential to impact the field by identifying new, sustainable models for how public health practitioners and academics can work together to meet common goals, increase use of EBPPs, and make efficient use of limited resources.

## Data Availability

Not applicable.

## Abbreviations

EBPPs: Evidence-based programs and policies
LHD: Local health department
AHD: Academic-public health department
EPIS: Exploration, Preparation, Implementation, and Sustainment
D4D: Designing for Dissemination
